# Evaluating Clonal Expansion of HIV-Infected Cells: Optimization of PCR Strategies to Predict Clonality

**DOI:** 10.1371/journal.ppat.1005689

**Published:** 2016-08-05

**Authors:** Sarah B. Laskey, Christopher W. Pohlmeyer, Katherine M. Bruner, Robert F. Siliciano

**Affiliations:** 1 Department of Medicine, Johns Hopkins University School of Medicine, Baltimore, Maryland, United States of America; 2 Howard Hughes Medical Institute, Baltimore, Maryland, United States of America; Vaccine Research Center, UNITED STATES

## Abstract

In HIV-infected individuals receiving suppressive antiretroviral therapy, the virus persists indefinitely in a reservoir of latently infected cells. The proliferation of these cells may contribute to the stability of the reservoir and thus to the lifelong persistence of HIV-1 in infected individuals. Because the HIV-1 replication process is highly error-prone, the detection of identical viral genomes in distinct host cells provides evidence for the clonal expansion of infected cells. We evaluated alignments of unique, near-full-length HIV-1 sequences to determine the relationship between clonality in a short region and clonality in the full genome. Although it is common to amplify and sequence short, subgenomic regions of the viral genome for phylogenetic analysis, we show that sequence identity of these amplicons does not guarantee clonality across the full viral genome. We show that although longer amplicons capture more diversity, no subgenomic region can recapitulate the diversity of full viral genomes. Consequently, some identical subgenomic amplicons should be expected even from the analysis of completely unique viral genomes, and the presence of identical amplicons alone is not proof of clonally expanded HIV-1. We present a method for evaluating evidence of clonal expansion in the context of these findings.

## Author Summary

Although antiretroviral therapy effectively blocks HIV-1 replication, the virus persists indefinitely in a reservoir of latently infected cells. This reservoir is a major barrier to HIV-1 cure. Recent studies have identified the proliferation of latently infected cells as a mechanism that may contribute to the lifelong persistence of HIV-1. In contrast with cellular proliferation, viral replication is highly error-prone; therefore, the detection of identical viral genomes in distinct host cells provides evidence that those cells are the progeny of an infected cell that underwent clonal expansion. For this reason, the accurate and reliable identification of identical HIV-1 genomes derived from distinct cells is critical for understanding HIV-1 persistence and advancing cure research. Studies of HIV-1 clonal expansion often present clonality of short, subgenomic sequences as evidence for the clonality of full viral genomes. In this study, we quantified the relationship between sequence identity in short regions and sequence identity of near-full-length genomes to demonstrate that no subgenomic region completely captures the diversity of the full viral genome. Consequently, identical subgenomic sequences are not proof of identical full-length HIV-1 genomes. In the context of these findings, we developed a method to evaluate identical subgenomic sequences as possible evidence for clonal expansion.

## Introduction

The HIV-1 virion carries two copies of a 9.7 kb RNA viral genome, which is reverse transcribed to DNA and integrated into the genome of a host cell during infection. Although combination antiretroviral therapy (ART) can suppress HIV-1 plasma viremia indefinitely to a level below the clinical limit of detection, the virus persists for decades in a latent reservoir composed of resting memory CD4^+^ T cells carrying integrated viral genomes, known as proviruses [1–4]. The development, composition, and plasticity of this latent reservoir, which presents a major barrier to the cure of HIV-1 infection, are all active areas of investigation [5,6].

An emerging body of research identifies the proliferation of latently infected CD4^+^ T cells as a possible mechanism for the persistence—and perhaps expansion—of the latent reservoir. Because viral replication is a low-fidelity process, expansion of the reservoir through cellular proliferation can be distinguished from expansion through *de novo* infection events by the presence of identical HIV-1 genomes with identical sites of integration into the host cell genome in distinct cells [7–9].

In part due to the substantial diversity of HIV-1 even within a single infected individual, generating full-length sequences of individual proviruses can be prohibitively expensive and labor intensive. Instead, it is common to sequence short PCR amplicons covering less than 3000 base pairs [7,8,10–18]. While these smaller, subgenomic amplicons contain sufficient sequence information to inform phylogenetic analysis, they do not capture the total diversity–in this case, defined as the proportion of non-clonal sequences—present in full-length viral genomes from the same sample. On the contrary, the sequence diversity in a subgenomic region is best understood as a minimum estimate of the total sequence diversity in the sample analyzed. Without comparing full-length HIV-1 genomes, it is impossible to determine whether two proviruses with identical sequence over a subgenomic amplicon are also identical over the remainder of the viral genome.

This inherent overestimation of viral clonality when comparing short, subgenomic sequences is of critical importance in the investigation of clonal expansion of latently infected cells as a mechanism of HIV-1 persistence. A number of studies have identified identical viral sequences in independent samples from a single subject [7,8,13,17–20]. These identical sequences may reflect the expansion of latently infected CD4^+^ T cells *in vivo*. However, when the identical sequences analyzed cover only a short fragment of the viral genome, they may also represent distinct infection events with viral genomes that happen to differ only in areas of the genome that were not analyzed. This distinction underscores the importance of understanding the relationship between the sequence diversity in subgenomic PCR amplicons and that of full viral genomes.

The goal of this study was to identify which short PCR amplicons—if any—capture the total diversity of full-length genomes in a sample. We analyzed near-full-length HIV-1 sequences available from previous studies; importantly, we specifically characterized intra-subject diversity. We considered how length, genomic position, and sample type contribute to the likelihood that a given subgenomic amplicon will include enough information to differentiate unique, full-length HIV-1 sequences. Given these findings, we characterized and evaluated eight PCR primer sets used in previously published studies of HIV-1 diversity for their ability to differentiate full-length viral sequences. We showed that subgenomic sequences are contextualized by the primer set used to generate them, and we present here a strategy for the evaluation of sequence data in the context of specific primer sets.

## Results

### Data sets and processing

We analyzed data sets containing between 5 and 121 (mean = 20.7, median = 9) unique, near-full-length HIV -1 sequences from a total of 31 subjects. None of the sequences analyzed cover the 5’ LTR, and all sequences are fully characterized at a minimum from positions 2000 through 8000 of the HXB2 reference genome. These sequences represent five different sample types: 1) proviral DNA from the resting CD4^+^ T cells of subjects who initiated suppressive ART during acute HIV-1 infection, designated “Acute treated–DNA”; 2) proviral DNA from the resting CD4^+^ T cells of subjects who initiated suppressive ART during unspecified stages of chronic HIV-1 infection, designated “Chronic treated–DNA”; 3) proviral DNA isolated from quantitative viral outgrowth assay (VOA) [21] wells negative for p24 antigen, representing resting CD4^+^ T cells that were not induced to produce replication-competent virus after stimulation with phytohemagglutinin, designated “VOA–DNA”; 4) genomic viral RNA isolated from the plasma of viremic subjects over a series of longitudinal time points, designated “Longitudinal–RNA”; and 5) genomic viral RNA isolated from the plasma of subjects during acute HIV-1 infection, designated “Acute–RNA.” The data sets were aligned to the HXB2 reference HIV-1 genome and processed to remove repeat sequences. Sequences were characterized as repeats if they were identical or differed only at ambiguously sequenced positions for all nucleotides sequenced. Thus, every full-genome sequence is unique in the alignments analyzed below. Details about sequence data sets and their sources are shown in Table 1 and S1 Table. We have shown that our results are not sensitive to the specific alignments; that is, the results are equivalent for different but equally probable alignments of the same sequences (S1 Fig). For this study, only alignments including at least five unique, near-full-length HIV-1 genome sequences from the same individual were considered.

**Table 1:** Sources and characteristics of sequence alignments analyzed to generate clonal prediction scores.

Sample	Source	Reference	PMID	Number of subjects	Sequences per subject (range)
**Acute treated–DNA**	DNA from resting CD4^+^ T cells of subjects treated during acute infection	Bruner *et al*., 2016, Nat. Med. [28]	-	6	7–14
**Chronic treated–DNA**	DNA from resting CD4^+^ T cells of subjects treated during chronic infection	Bruner *et al*., 2016, Nat. Med. [28]	-	5	8–13
**VOA–DNA**	DNA from p24 antigen-negative quantitative viral outgrowth assay [21] culture wells	Ho *et al*., 2013, Cell. [22]	24243014	5	6–12
**Longitudinal–RNA**	Plasma RNA from 2–10 longitudinal samples per subject	Herbeck *et al*., 2011, J. Virol. [15]	21593162	6	28–121
**Acute–RNA**	Plasma RNA collected during acute infection	Salazar-Gonzalez *et al*., 2009, J. Exp. Med. [14]	19487424	9	5–14

**Fig 1:**
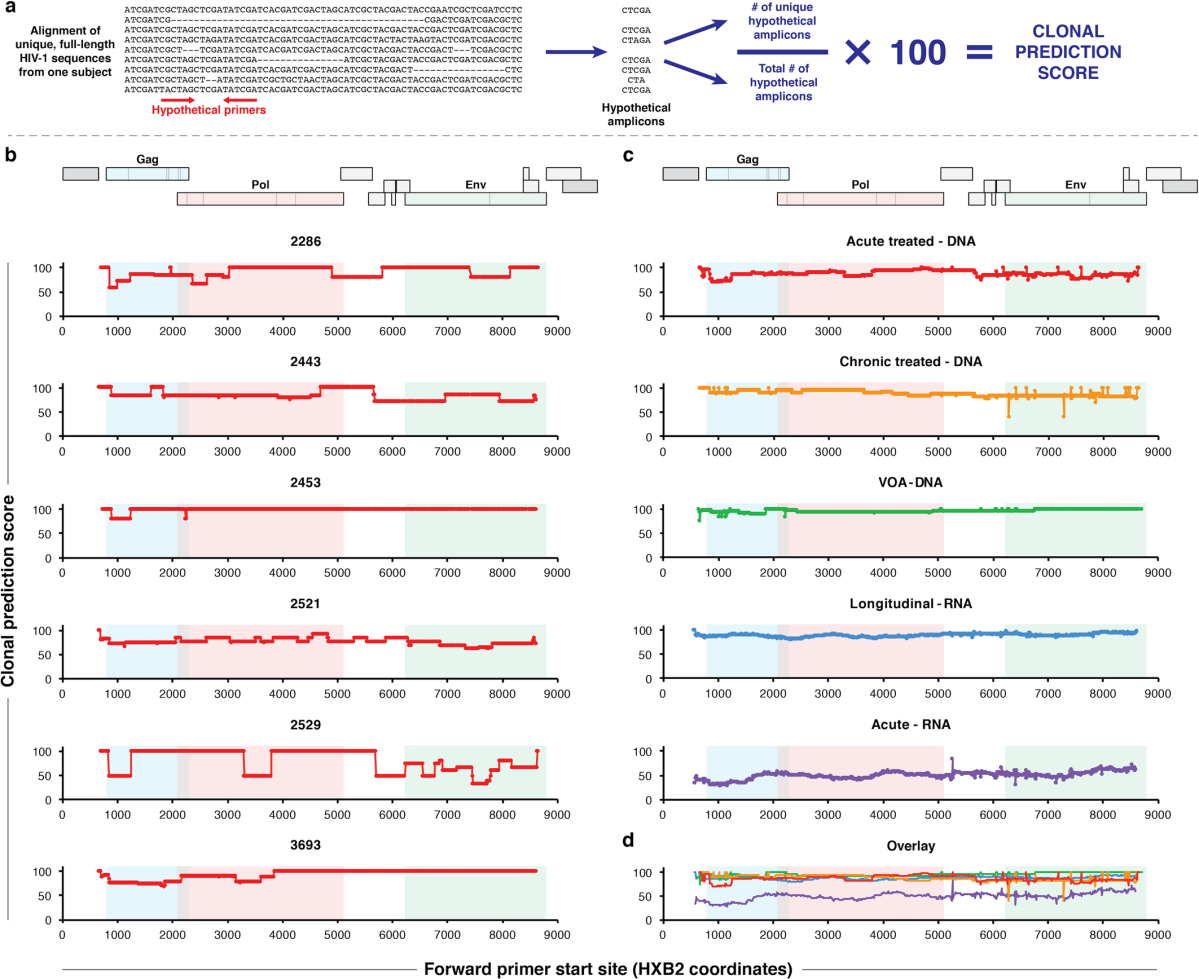
Clonal prediction scores of 1 kb amplicons spanning the HIV-1 genome. (**a**) Schematic of algorithm to calculate clonal prediction score (CPS), which quantifies the proportion of unique sequences in an alignment that are correctly identified as unique using the amplicons produced by a specific primer set. (**b**) CPS of 1 kb-wide amplicons spanning the HIV-1 genome for six Acute treated–DNA subjects. (**c**) CPS of 1 kb-wide amplicons spanning the HIV-1 genome, averaged over all subjects in five different sample categories (Table 1). (**d**) Overlay of the five plots in part **c**. Schematics of the HIV-1 genome are aligned to the charts in parts **b** through **d** to highlight viral gene locations. The amplicons in parts **b** through **d** are defined with reference to the HXB2 genome.

Because our goal was to characterize intra-subject diversity, we analyzed the alignments from each subject individually. Fig 1A shows a schematic of our analytic procedure. We considered a series of hypothetical primer pairs defining subgenomic PCR amplicons. Each of these primer sets was evaluated against each subject sequence alignment. We assumed that a sequence could be amplified by a primer set if the sequence aligned to the HXB2 reference genome without any gaps in either primer site.

For each sequence alignment, we considered the amplicons that would be produced by a PCR with a given hypothetical primer set, discarding the sequences that would not be amplified by that primer set due to insertions or deletions overlapping the primer binding sites. We chose to consider only PCR-amplifiable sequences to maximize the practical value of our metric for the analysis of sequence data sets generated using PCR-based protocols. We defined the *clonal prediction score* (CPS) of the primer set as the number of unique amplicons produced, divided by the total number of amplicons produced, and multiplied by 100 (Fig 1A). In words, the CPS of a primer set with respect to an alignment is defined as the percentage of sequences in the alignment that would be correctly identified as unique using only the sequence region amplified by those primers. Each full genome in these alignments is unique, and primer sets that produce a unique amplicon for every amplifiable sequence in the alignment have a CPS of 100.

These parameters define a maximal CPS of 100 and a minimum possible CPS of 100/*N*, where *N* is the number of amplified sequences. When a primer set will not amplify any of the sequences in an alignment, the CPS is undefined for that primer set with respect to that alignment. Undefined CPS values are excluded from the figures described below. Importantly, the precision of the CPS is limited by the number of sequences amplified by a primer set. For example, a primer set that correctly distinguishes 5 of 5 amplicons would have the same perfect CPS = 100 as a primer set that correctly distinguishes 100 of 100 amplicons. If a new unique sequence were added to the alignment and incorrectly identified as clonal, the CPS values in these two cases would change to 83 and 99, respectively. For this reason, the empirically perfect CPS = 100 may be more precisely described as CPS > 100*N/(N+1). This example emphasizes why alignments containing more sequences lead to greater precision in CPS values. For clarity, we have plotted maximal CPS values at CPS = 100 in the figures described below.

### Substantial variation in CPS among subjects

To investigate how CPS varies across the HIV-1 genome and across infected individuals, we calculated CPS values for hypothetical 1 kb amplicons spanning the HIV-1 genome at 10 bp intervals. These amplicons were defined by hypothetical 10 bp forward and reverse primers, and we have shown that the results of the following analysis are not sensitive to the choice of primer length (S2 Fig). While the amplicons defined by these hypothetical primers have a length of 1 kb in the HXB2 reference genome, they may have different lengths in sequences containing insertions or deletions between the primer sites. Along with sequence differences, length polymorphisms can be used to differentiate unique sequences.

The results of this analysis for six Acute treated–DNA samples (Table 1) are shown in Fig 1B. There was substantial variation among subjects, suggesting that a primer set optimized to differentiate sequences in one sample may not be optimal in another sample from a different subject. For subject 2453, almost every 1 kb amplicon contained sufficient variation to differentiate all amplified sequences, but the other five subjects had CPS values below 100 for amplicons spanning large portions of the genome. For subject 2521, less than one percent of all possible 1 kb amplicons had a perfect CPS of 100. That is, almost every 1 kb amplicon would incorrectly classify unique genomic sequences from subject 2521 as identical. There is no relationship between these patterns and the number of sequences analyzed for each subject (S3 Fig and S1 Table).

Plots of CPS across the genome for individual Chronic treated–DNA, VOA–DNA, Longitudinal–RNA, and Acute–RNA samples (Table 1) are shown in S3 Fig. The perfect CPS of 100 is much more common for proviral DNA than for plasma RNA; in Chronic treated–DNA and VOA–DNA samples, more than half of subjects had perfect CPS values for every 1 kb amplicon across the viral genome. These perfect CPS values are often found at locations in the genome where one or more sequences in the alignment contain deletions and cannot be amplified. In these cases, the total number of sequences detected by a primer set (the denominator in the CPS equation) is lower, and there are fewer amplicons to be differentiated. Although alignments containing only a few sequences do not lead to bias or inaccuracy in the CPS, the precision of the CPS calculation is correlated with the number of sequences in the alignment being analyzed. In the compilation of our data set, we chose to include only subjects for whom at least five unique, near-full-length sequences had been characterized, but the precision of these results would be improved by the collection and inclusion of more sequences per subject.

The dramatic variation in CPS among different subjects is seen in all sample types assayed except Longitudinal–RNA (S3 Fig). For Longitudinal–RNA samples, CPS is consistent between subjects and across the viral genome. This result reflects the high diversity of the HIV-1 quasispecies during chronic infection.

### Lack of genomic hotspots for CPS across sample types

To determine whether optimized primer sets for the identification of clonal HIV-1 sequences should be located in specific regions of the viral genome, we plotted the CPS for hypothetical 1 kb amplicons spanning the HIV-1 genome, averaged over all of the subjects within each sample group (Fig 1C). The top plot in Fig 1C shows the average of the six plots in Fig 1B, and the other plots in Fig 1C show averages of the plots in S3 Fig. Fig 1D is an overlay of the five plots in Fig 1C. The purpose of these averaged scores was to determine whether any region of the viral genome yields consistently higher or lower CPS than other regions across different subjects or sample types.

CPS values averaged over several subjects are relatively consistent across the viral genome for all five sample types evaluated. In the Acute treated–DNA and Chronic treated–DNA samples, CPS values appear slightly higher for the 5’ half of the genome than for the 3’ half. In contrast, the CPS for VOA–DNA samples is highest at the 3’ end of the genome. Importantly, these general trends are not representative of individual subjects, *e*.*g*., Acute treated–DNA subject 3693 has a perfect 100 CPS only for amplicons toward the 3’ end of the viral genome (Fig 1B).

The Acute–RNA samples stand out as having lower CPS values across the viral genome than the other sample types (Fig 1D), indicating that the sequences in these alignments differ at relatively few places in the genome. This finding is consistent with the biological characteristics of the Acute–RNA sample type; these sequences contain low genetic diversity because they represent plasma collected during acute HIV-1 infection, before the viral quasispecies has expanded and developed the sequence diversity characteristic of chronic infection. In most individuals, HIV-1 infection is initiated by a single transmitted founder virus that expands into a diverse quasispecies over the course of untreated infection [22,23].

Importantly, these results show that there is no optimal region of the genome best suited for differentiating unique sequences in a subject- or sample type-independent manner.

### Effect of amplicon length on CPS

**Fig 2:**
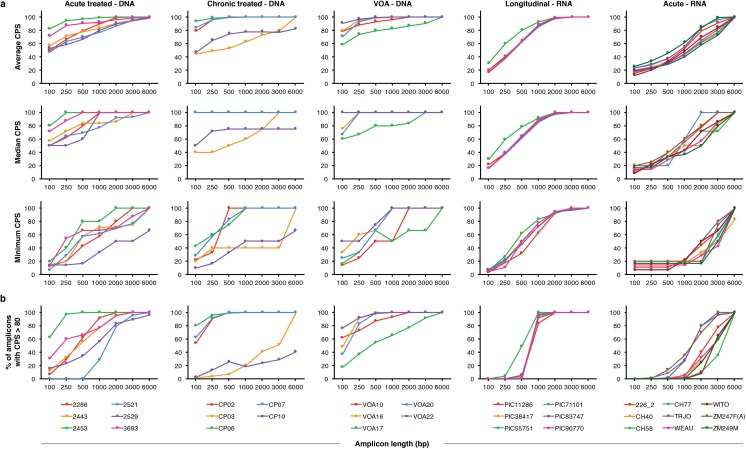
Relationship between amplicon length and CPS. Summary statistics describing all amplicons of a given length, spanning the HXB2 reference genome at 10 bp intervals. Amplicons with undefined CPS were not included in these summary statistic calculations. (**a**) Average, median, and minimum of the CPS values for every amplicon of a specified length spanning the viral genome. Summary statistics are shown for each subject and grouped by sample type. (**b**) Proportion of all of the amplicons of a specified length that have CPS values above 80.

To determine the effect of amplicon length on CPS, we calculated the CPS for hypothetical amplicons of different lengths spanning the HXB2 genome. We summarized the analysis of all amplicons of a given length in three ways, by taking the average, median, and minimum CPS over all amplicons of that length across the genome (Fig 2A). For example, all of the points plotted in the top chart from Fig 1B are averaged together to yield the subject 2286, 1000 bp data point in the Acute treated–DNA Average CPS plot in Fig 2A. Individual results for the 31 subjects are grouped by sample type.

For every subject, regardless of sample type, longer amplicons yield higher CPS. This is unsurprising, as a longer amplicon should be more likely than a short amplicon to contain sequence diversity. However, the precise relationship between amplicon length and CPS varies dramatically by sample type. In proviral DNA samples, CPS increases with amplicon length but varies among subjects. For very short amplicons between 100 bp and 500 bp, average and median CPS can range from about 40 to a perfect 100. For very large amplicons of 6 kb, the average CPS is greater than 80 and the median CPS is 100 for almost every proviral DNA sample. Again, there is no correlation between CPS and the number of amplicons in a sequence alignment (Fig 2 and S1 Table).

In DNA from subjects treated during chronic infection, the relationship between amplicon length and CPS is similar for three of five subjects. CPS values are high for the three Chronic treated–DNA samples that cluster together in Fig 2; all three subjects have a perfect CPS of 100 across the viral genome for amplicons as short as 1 kb. Subject CP03 is believed to have initiated therapy early in chronic infection, which may explain the similarity of that sample to the Acute treated–DNA samples. Subject CP10 illustrates an important limitation of any sequence-based analysis. Two of the sequences in the CP10 alignment differ by a single nucleotide. These sequences likely represent identical genomes that differ because of an error generated during PCR, but they were considered to be unique for this analysis. Because the error rate of PCR using high-fidelity polymerases is of a much lower magnitude than HIV-1 sequence diversity, our results overwhelmingly reflect true viral diversity (rather than technical diversity) except in this exceptional case of PCR error in identical genomes.

In plasma RNA samples, the relationship between amplicon length and CPS is direct and consistent across subjects. Unlike with the proviral DNA samples, the average and median CPS for plasma RNA can be lower than 20 for 100 bp amplicons. These low CPS values indicate alignments with many detectable sequences but insufficient sequence diversity to distinguish them using small amplicons.

The relationship between amplicon length and CPS is direct and linear for Longitudinal–RNA samples. There is much less inter-subject variation in this relationship for Longitudinal–RNA than for any of the other sample types. Importantly, even the minimum CPS increases predictably with amplicon length for these samples. For all Longitudinal–RNA samples, the average CPS is greater than 95 for 2 kb amplicons, and even the minimum CPS for a 2 kb amplicon is greater than 90. This contrasts with the other sample types, where average CPS increases predictably with amplicon length but minimum CPS is variable.

Fig 2A shows that most short PCR amplicons are limited in their capacity to distinguish unique HIV-1 genomes, and that the goal of a single short amplicon with a perfect CPS of 100 in a variety of subjects and sample types is unattainable. Instead, we evaluated how many amplicons achieve high—but not necessarily perfect—CPS values. Fig 2B shows the proportion of amplicons of a given length with CPS > 80. The relationship between amplicon length and the frequency of amplicons with CPS > 80 is largely consistent with the results shown in Fig 2A but emphasizes the inter-subject differences in CPS patterns.

### Variation in PCR coverage with sample type and amplicon length

**Fig 3:**
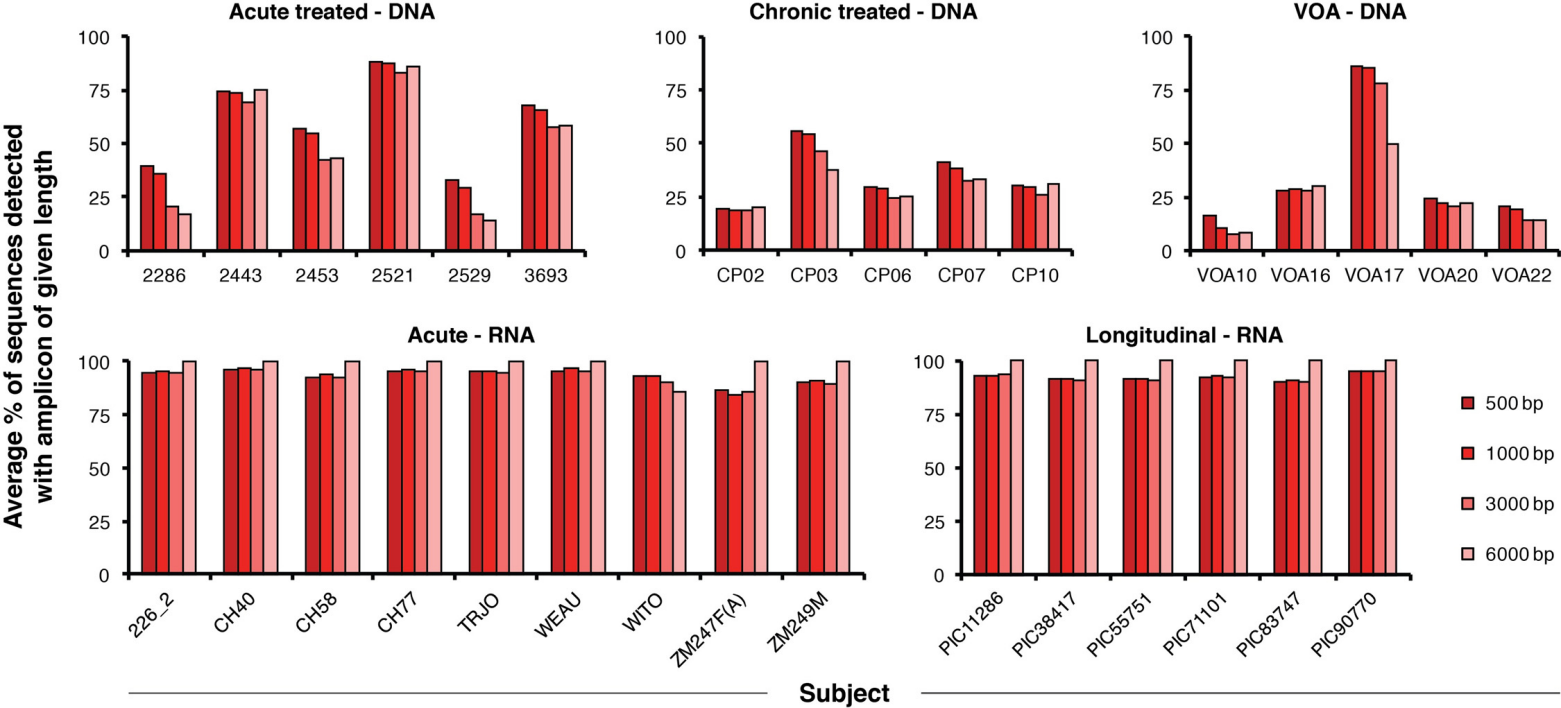
Relationship between amplicon length and PCR coverage. Percentage of sequences in each alignment that would be detectable by PCR, averaged over all amplicons of a specified length spanning HXB2 positions 2000 through 8000 at 10 bp intervals. Results are shown for 31 subjects and grouped by sample type (Table 1).

Importantly, CPS is undefined for a primer set that will not amplify any of the sequences in a given alignment. The summary statistics in Fig 2 describe only the primer sets with defined CPS for each subject. In other words, the CPS values shown in Fig 2 represent only the primer sets for which at least one sequence in the alignment is detectable. We calculated PCR coverage, or the fraction of sequences in each alignment that would be detectable by PCR, averaged over all amplicons of a given length between HXB2 coordinates 2000 and 8000 (Fig 3). We chose this region because it is fully characterized for every sequence in our data set, and so the data in Fig 3 reflect the presence of internal deletions in the sequences rather than missing sequence data. As described above, we considered amplicons defined by hypothetical primer sets spanning the region at 10 bp intervals.

The PCR coverage reflects the overall percentage of the viral genome lost to internal deletions in each sample. This value is consistent across subjects in plasma RNA samples (Fig 3), for all of which the proportion of detectable amplicons approaches 100%, especially with very large 6 kb amplicons. Plasma RNA represents viral genomes capable of producing all of the viral proteins necessary to generate a functional virion and therefore does not contain large internal deletions. In contrast, proviral DNA samples are much less likely to be detected by PCR because of the high frequency of large internal deletions in archived proviral genomes [24]. There is substantial variation in PCR coverage for proviral DNA samples among different subjects and across sample types. On average, Acute treated–DNA sample sequences are more likely to be detected by PCR than Chronic treated–DNA or VOA–DNA sample sequences.

The results shown in Fig 3 have implications for primer design independent of CPS. In proviral DNA samples, the results of PCR-based analyses may vary dramatically with the choice of primers because different sequences in the sample can be intact or deleted in different regions of the viral genome; bias may be introduced by the specific selection of only the sequences that happen to be intact in a particular region.

### CPS of previously published amplicons

**Fig 4:**
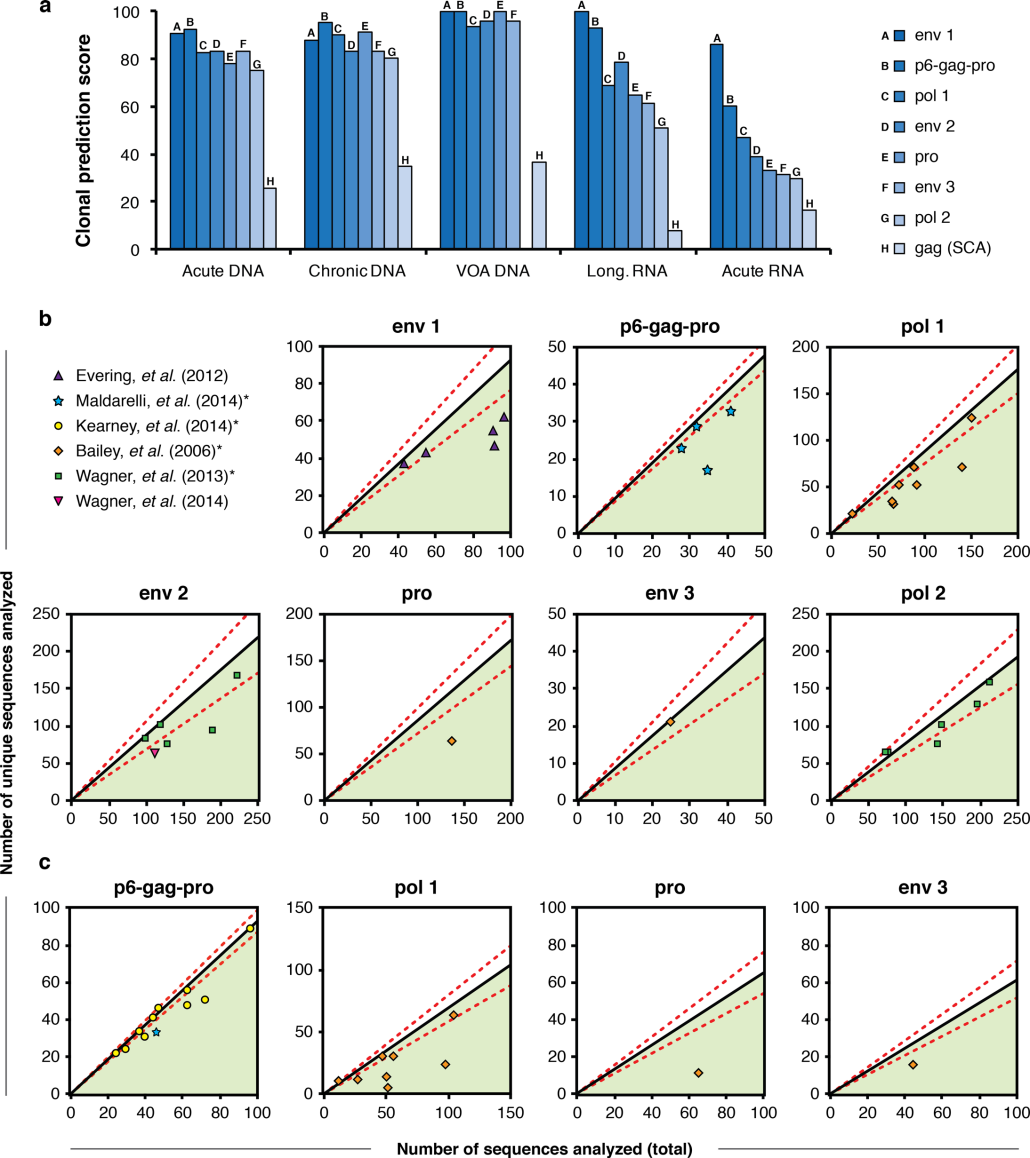
CPS of previously published amplicons. (**a**) CPS for each of the primer sets listed in Table 2, averaged over all subjects within each sample type (Table 1). (**b** and **c**) Evaluation of previously published phylogenetic trees in the context of the CPS of the primer sets used to generate those trees. Part **b** shows proviral DNA samples and part **c** shows plasma RNA samples. Black diagonal lines show the relationship between the number of sequences collected and the number expected to be unique for each primer set listed in Table 2, as an estimate of the background signal level (assuming no clonality); the slopes of these lines are equal to the CPS values in part **a** divided by 100. The dotted red lines were calculated as the black lines plus or minus one standard deviation in CPS. Each plotted point indicates the actual number of total sequences and unique sequences present in a previously published phylogenetic tree. Phylogenetic trees containing both proviral DNA and plasma RNA sequences were counted separately for the two sequence types and plotted separately in parts **b** and **c**. Points plotted far below the black line (green-shaded region) indicate trees with more clonality than would be expected by chance from a sample of unique HIV-1 genomes. *References in which hypermutated sequences were not explicitly included in phylogenetic trees.

We calculated the CPS for eight primer sets used in published studies of HIV-1 diversity, evolution, and clonal expansion (Table 2) with respect to the sequence alignments in our data set. The results, averaged over all subjects for each sample type, are presented in Fig 4A. Consistent with the results in Fig 2, larger amplicons typically have higher CPS values, and this relationship is most distinct in plasma samples. Importantly, in most of the samples shown, none of the published primer sets have a perfect CPS of 100. Consequently, in any analysis of sequences derived using these primers, some unique full-length genomes will be misrepresented as identical amplicons.

**Table 2:** Amplicons from published studies of HIV-1 diversity, evolution, and clonal expansion.

Primer set	Reference	PMID	Amplicon length (bp)	Amplicon HXB2 coordinates	F primer	R primer
**env 1**	Evering *et al*., 2012, PLoS Pathog. [16]	22319447	2948	5956–8903	TTAGGCATCTCCTATGGCAGGAAGAAG	GTCTCGAGATACTGCTCCCACCC
**p6-gag-pro**	Palmer *et al*., 2005, J. Clin. Microbiol. [12]	15635002	1565	1870–3434	GAGTTTTGGCTGAAGCAATGAGCC	TTAGTGGTACTACTTCTGTTAGTGTT
**pol 1**	Bailey *et al*., 2006, J. Virol. [13]	16775332	664	2598–3261	AYGGCCCAARAGTYAAAC	TTATCAGGATGGAGYTCA
**env 2**	Delwart *et al*., 1993, Science [10]	8235655	667	7001–7667	CTGTTAAATGGCAGTCTAGC	CACTTCTCCAATTGTCCCTCA
**pro**	Bailey *et al*., 2006, J. Virol. [13]	16775332	537	2056–2592	TGAAAGATTGTACTGAGAGACAGG	CCTGGCTTTAATTTTACTGGTACAG
**env 3**	Bailey *et al*., 2006, J. Virol. [13]	16775332	471	1059–7529	ACAATGCTAAAACCATAATAGT	CATACATTGCTTTTCCTACT
**pol 2**	Wagner *et al*., 2013, J. Virol. [17]	23175380	416	2200–2615	CTCCCCCTCAGAAGCAGGAGCCGATAGACAAGGAACTGTATCC	GGATGGCCCAAAAGTTAAAC
**gag (SCA)**	Palmer *et al*., 2003, J. Clin. Microbiol. [11]	14532178	79	1298–1376	CATGTTTTCAGCATTATCAGAAGGA	TGCTTGATGTCCCCCCACT

### Using CPS to quantify false-positive clonality

The analysis of subgenomic amplicons to characterize HIV-1 genomes cannot provide definitive evidence of clonality in a sample, even when identical sequences are detected. The CPS for a primer set indicates how much false-positive clonality is found by that primer set in alignments of unique genomes. Thus the CPS for alignments of unique genomes can be understood as an estimate of the background signal expected when using a given primer set to evaluate clonality. We used the CPS of published primer sets to estimate the presence or absence of true clonality in published phylogenetic trees. This analysis is described in detail in the Methods section.

In short, we plotted the relationship between the total number of sequences analyzed and the number of unique sequences detected (Fig 4B and 4C). The CPS of a primer set is represented as a black line on these plots, and red dashed lines indicate the CPS plus or minus one standard deviation. We then plotted phylogenetic trees from six previously published studies [7,8,13,16–18] as points on the same axes. Each plot in Fig 4B and 4C shows a different primer set, and each point represents a single phylogenetic tree generated using that primer set. Fig 4B shows results for trees composed of proviral DNA samples, and Fig 4C shows trees composed of plasma RNA samples. Trees containing both RNA and DNA were separated and evaluated individually; these trees are represented by separate points in Fig 4B and Fig 4C. The distance between the lines (CPS, background signal estimate) and the points (sample data) demonstrate the amount of true clonality likely to be present in a sample.

Importantly, the black lines in Fig 4B and 4C do not lie along the x = y axis. For every primer set defining a subgenomic amplicon, even in a sample composed entirely of unique HIV-1 genomes, some false-positive clonal amplicons are likely to be detected, causing the slope of the black line to deviate from 1. The expected number of false positive identical amplicons (y-axis) increases proportionally with the total number of amplicons sequenced (x-axis).

Points that lie on the black lines are consistent with the null hypothesis that there are no clonally expanded HIV-1 genomes in the sample. Points that fall below the black line suggest clonal expansion, *i*.*e*., the presence of more identical amplicons than would be expected in a sample composed of unique viral genomes. We did not see evidence of points plotted well above the black line, which would have indicated greater sequence diversity than was present in the alignments used to generate this model. This observation serves as validation of the data sets used to train our model.

Studies in which trees do not include hypermutated sequences are marked with an asterisk. Hypermutated sequences are easily distinguishable even using short amplicons because of their tremendous diversity (S4 Fig). For this reason, the removal of hypermutated or otherwise defective sequences from an alignment will artificially deflate the ratio of unique sequences to total sequences detected. In other words, for trees that have been curated to remove hypermutated sequences, the true location of the points plotted in Fig 4B and 4C is expected to fall closer to the black line. Due to the mechanisms of APOBEC-mediated hypermutation and HIV-1 reverse transcription, the rate of hypermutation is not constant across the viral genome [25,26]; the relationship between CPS and genome location in hypermutated sequences is shown in S4B Fig.

The analysis shown in Fig 4 provides a method for evaluating both primer sets and sequence data. A primer set with high CPS and minimal variation in CPS across samples provides the most powerful background estimate to enable the confident interpretation of sequence data. And regardless of the primers used to generate sequences, phylogenetic trees and sequence alignments can only be understood properly when interpreted in the context of the CPS for the primer set used to generate them.

## Discussion

In this study, we defined the CPS as a metric for how well a subgenomic amplicon differentiates unique HIV-1 genomes. We calculated CPS values for hypothetical amplicons of varying sizes across the HIV-1 genome to investigate the contribution of size and location to the capacity of an amplicon to distinguish unique genomes. Finally, we calculated the CPS values for commonly used primer sets and used them to estimate the background level of clonality in phylogenetic trees generated with those primer sets.

The primary goal of this study was to identify PCR amplicon(s) best suited to differentiate unique, full-length HIV-1 genomes. We found compelling evidence that no single, subgenomic amplicon will be sufficient to distinguish HIV-1 genomes across a variety of sample types or subjects. However, we evaluated eight previously published primer sets and found that of those, a 1.5 kb amplicon in the p6-gag-pro region seems to be the best compromise between coverage, practicality, and CPS, a metric which describes the capacity of an amplicon to correctly identify unique sequences as unique.

We also identified a variety of best practices to maximize the validity of future studies of HIV-1 clonality in any sample type. Most importantly, researchers should always emphasize which region is being sequenced and explicitly consider the CPS of the amplicon(s) used. This context is essential for evaluating claims of clonal expansion, as it provides an estimate of the expected background signal against which sequencing results should be compared. We recommend against highlighting identical amplicons as evidence of full-genome clonality without comparing those results to an appropriate background estimate, and we emphasize that the only way to definitively demonstrate clonal expansion is to corroborate results with full-genome and integration site sequencing.

Importantly, all of the analyses presented here assume that the individual amplicons sequenced were collected independently using methods specifically designed for single-genome sequencing. Many studies are confounded by PCR resampling [27]; all sequences analyzed using the methods described here should be collected independently.

Furthermore, although some analyses call for curated sequence data, every full, uncurated data set should be made available. Phylogenetic trees presented to highlight clonally expanded populations should not be curated to remove replication-incompetent sequences. As described above, the removal of hypermutated sequences from a data set artificially inflates the proportion of clonal sequences in that data set. When evaluating alignments or trees against the background CPS of the amplicon sequenced, it is necessary to analyze every sequence collected.

To aid researchers in the analysis of new data sets with different amplicons than those described here, we have published a Web tool available at http://silicianolab.johnshopkins.edu/cps. This tool computes the CPS for user-specified primer sets and performs a comparison with user-entered values to characterize phylogenetic trees or alignments of amplicons in the context of appropriate background signal estimates.

Whether using this CPS analysis or any other method, it is impossible to conclusively prove or disprove the clonality of full-length viral genomes using only the sequence of a subgenomic amplicon. However, because information about the region amplified is available, it is essential to consider this context when interpreting data. These implications are no less relevant for phylogenetic studies in fields beyond HIV persistence; whenever the results and interpretation of a phylogenetic analysis may be impacted by the choice of amplicon sequenced, it is critical to consider that impact explicitly in the evaluation and presentation of data. This CPS analysis is intended not as a definitive arbiter of full-sequence clonality but as a tool to quantify the context provided by primer location and inform the interpretation of sequence data.

Some important limitations of this study suggest additional considerations for future research. We have considered amplicon sequences on a binary scale; either sequences are correctly identified as unique, or they are not. We have not considered the distribution of sequence clonality. For example, in an amplicon alignment with twenty total sequences and only ten sequences correctly identified as unique, we did not distinguish between cases where the ten clonal sequences are identical to each other and cases where they represent duplicates of several other sequences. The case where all clonal sequences are identical may or may not be more likely to represent full-genome clonality; the quantification of that likelihood is beyond the scope of this study.

In this study, we took advantage of sequence alignments representing a variety of sample types. Importantly, the results of our analyses often varied dramatically, especially between plasma RNA HIV-1 genomes, which are typically intact, and proviral DNA genomes, which often contain large internal deletions. Although the CPS of a primer set is clearly correlated across sample types, the implications of our analysis for different sample types than those evaluated in this study are imprecise. We have also shown that even within a sample type, the variation in CPS among subjects can be extreme. Additionally, especially in the case of proviral DNA sequences containing deletions, the methods used to identify full-length genomic HIV-1 sequences in previous studies may have been more efficient for some sequences than for others. Any bias in the nature of the sequences used to define our CPS model may be reflected in our results. For these reasons, the model presented here and any future calculations of CPS would benefit from the inclusion of more full-length or near-full-length HIV-1 genome sequences and sample types collected in the future.

In summary, identical HIV-1 sequence fragments must be validated to demonstrate full-genome clonality conclusively. This validation can be achieved in a variety of ways. When technical constraints permit, additional regions of the genome can be sequenced. To demonstrate the clonality of proviral DNA sequences, the best validation is to sequence the associated integration site. Unvalidated sequences may be described as “identical throughout the region sequenced” but without further corroboration should not be described as clonally expanded. The web tool available at http://silicianolab.johnshopkins.edu/cps can be used to calculate CPS for the evaluation of new data or primer sets not described in this study.

## Methods

### Online tool

The online tool to calculate CPS can be found at http://silicianolab.johnshopkins.edu/cps. The tool is written in JavaScript, and the code is accessible at https://github.com/gitliver/HIVCPS. All sequences used for CPS analysis are available from GenBank.

### CPS plots

The CPS values in Fig 4A are similar across all three DNA sample types. To calculate the expected CPS of a given primer set as used to characterize a proviral DNA sample, we averaged the CPS of that primer set for the three DNA sample types. To calculate the expected CPS of a given primer set used to characterize a plasma RNA sample, we used the average CPS from the Longitudinal–RNA samples (Fig 4A). We chose not to include the Acute–RNA samples in this average because plasma virus from acutely infected individuals contains much less diversity than any other sample type, and its relevance to the analysis of other samples is minimal. The appearance of the Acute–RNA sample type as an outlier is evident in Figs 1, 2 and 4A.

For each primer set in Table 2, we used the average CPS values for proviral DNA and plasma RNA samples to calculate the proportion of false-positive clonal sequences that should be expected from sequencing subgenomic HIV-1 RNA or DNA amplicons. The relationship between the total number of amplicons sequenced and the expected number of correctly identified unique amplicons detected for each primer set is plotted as a black line in Fig 4B (proviral DNA) and 4c (plasma RNA). The slope of this line is the CPS divided by 100. The dashed red lines give a confidence interval representing one standard deviation in CPS values.

We counted the total number of sequences and the number of unique sequences in 53 different phylogenetic trees from six previously published studies [7,8,13,16–18]. The color and shape of the points indicate the study in which each tree was published.

## Supporting Information

S1 Table Number and subtype of unique sequences analyzed for each subject. (TIF)

S1 Fig Clonal prediction scores are equivalent for different, equally probable alignments of the same sequences. The Longitudinal–RNA sample sequences were aligned to the HXB2 reference genome in two different but equally probable alignments. CPS values were calculated for 1 kb amplicons spanning the viral genome at 10 bp intervals (see Fig 1). Parts **a** and **b** show that CPS values are equivalent for the two different alignments.
(TIF)

S2 Fig Clonal prediction scores of hypothetical amplicons are insensitive to the choice of hypothetical primer length. CPS values were calculated for 1 kb amplicons spanning the viral genome at 10 bp intervals. These amplicons were defined by hypothetical primers based on the HXB2 reference genome. The choice of hypothetical primer length used to define the amplicons characterized in Fig 1 is arbitrary; we show here that the results in Fig 1D are insensitive to variation in hypothetical primer length.
(TIF)

S3 Fig Clonal prediction scores of 1 kb amplicons spanning the HIV-1 genome for individual subject sequence alignments. CPS of 1 kb-wide amplicons spanning the HIV-1 genome for all subjects not shown in Fig 1B. The average of these plots over all subjects within each sample type are shown in Fig 1C.
(TIF)

S4 Fig Clonal prediction scores of hypothetical short amplicons used to characterize hypermutated or non-hypermutated sequences. CPS values were calculated separately for the hypermutated and non-hypermutated sequences from the same subjects. Results are shown for four subjects with ≥4 unique hypermutated sequences and ≥3 unique non-hypermutated sequences. (**a**) Average CPS values over all hypothetical amplicons of a given length spanning the viral genome are shown for the hypermutated-only and non-hypermutated-only alignments (see Fig 2). CPS values are higher for hypermutated sequences, indicating that hypermutated sequences are much easier to distinguish than non-hypermutated sequences using amplicons as small as 100 bp. (**b**) CPS of 200 bp-wide amplicons spanning the HIV-1 genome (see Fig 1) with hypermutated and non-hypermutated sequences evaluated separately. The top plot emphasizes locations in the genome where even hypermutated sequences are not always distinguishable by a 200 bp amplicon.
(TIF)
