# Supplementary figures and images for: Evaluating Clonal Expansion of HIV-Infected Cells: Optimization of PCR Strategies to Predict Clonality
Source: PLoS Pathog. 2016 Aug 5;12(8):e1005689. doi: 10.1371/journal.ppat.1005689 (PMC4975415; doi:10.1371/journal.ppat.1005689)

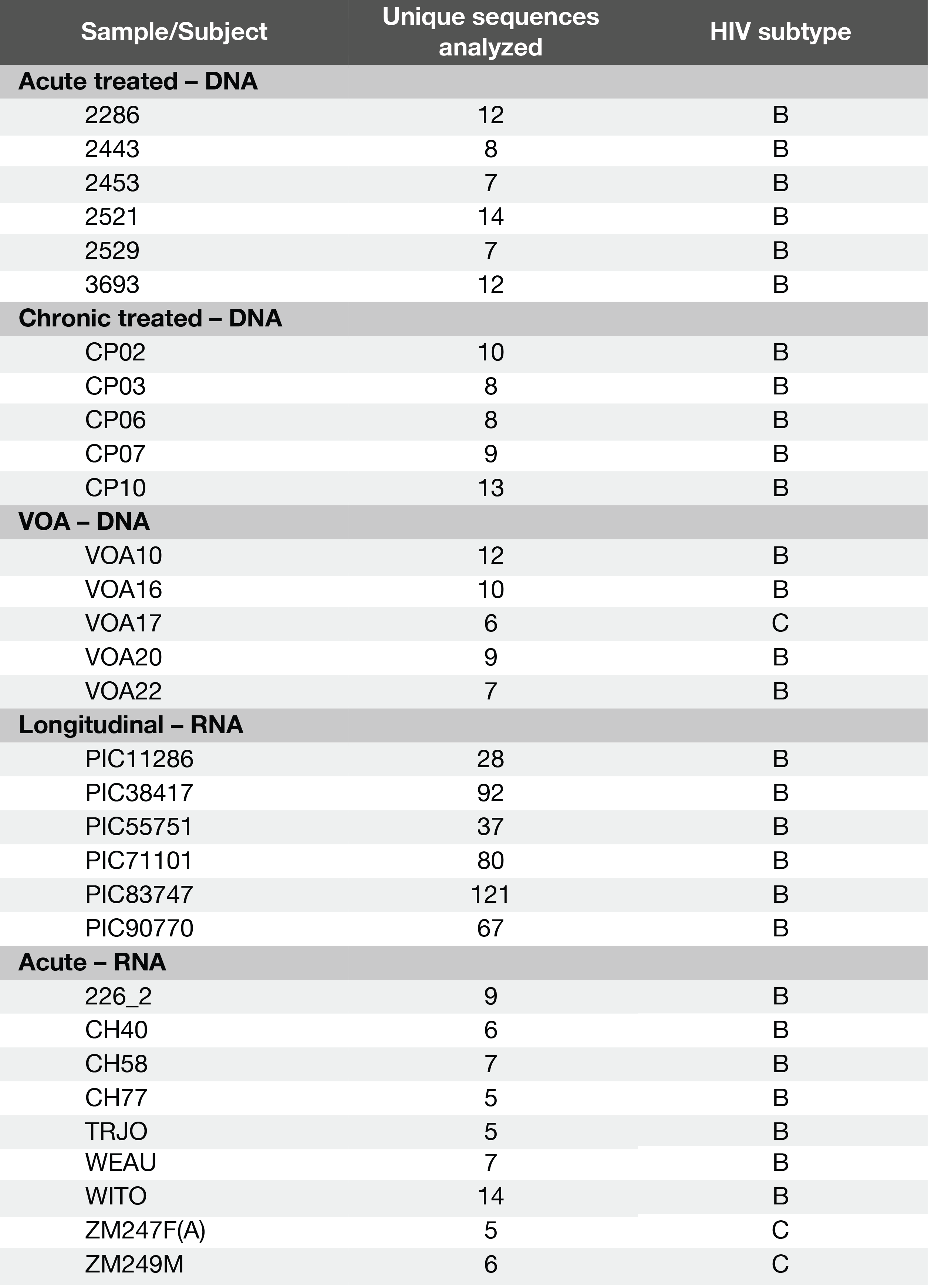

Supplement: S1 Table — (TIF) [file ppat.1005689.s001.tif]

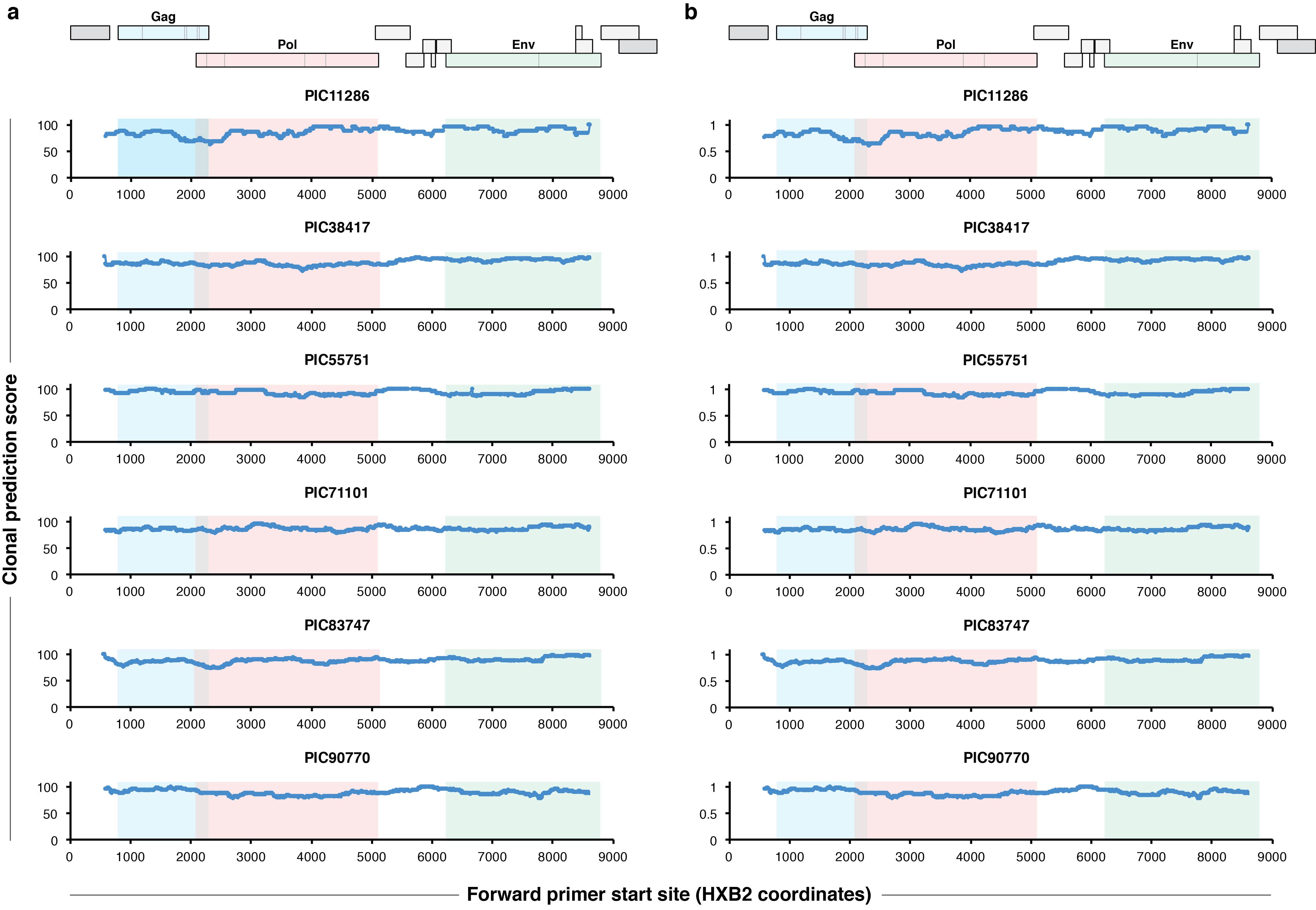

Supplement: S1 Fig — The Longitudinal–RNA sample sequences were aligned to the HXB2 reference genome in two different but equally probable alignments. CPS values were calculated for 1 kb amplicons spanning the viral genome at 10 bp intervals (see Fig 1). Parts a and b show that CPS values are equivalent for the two different alignments. (TIF) [file ppat.1005689.s002.tif]

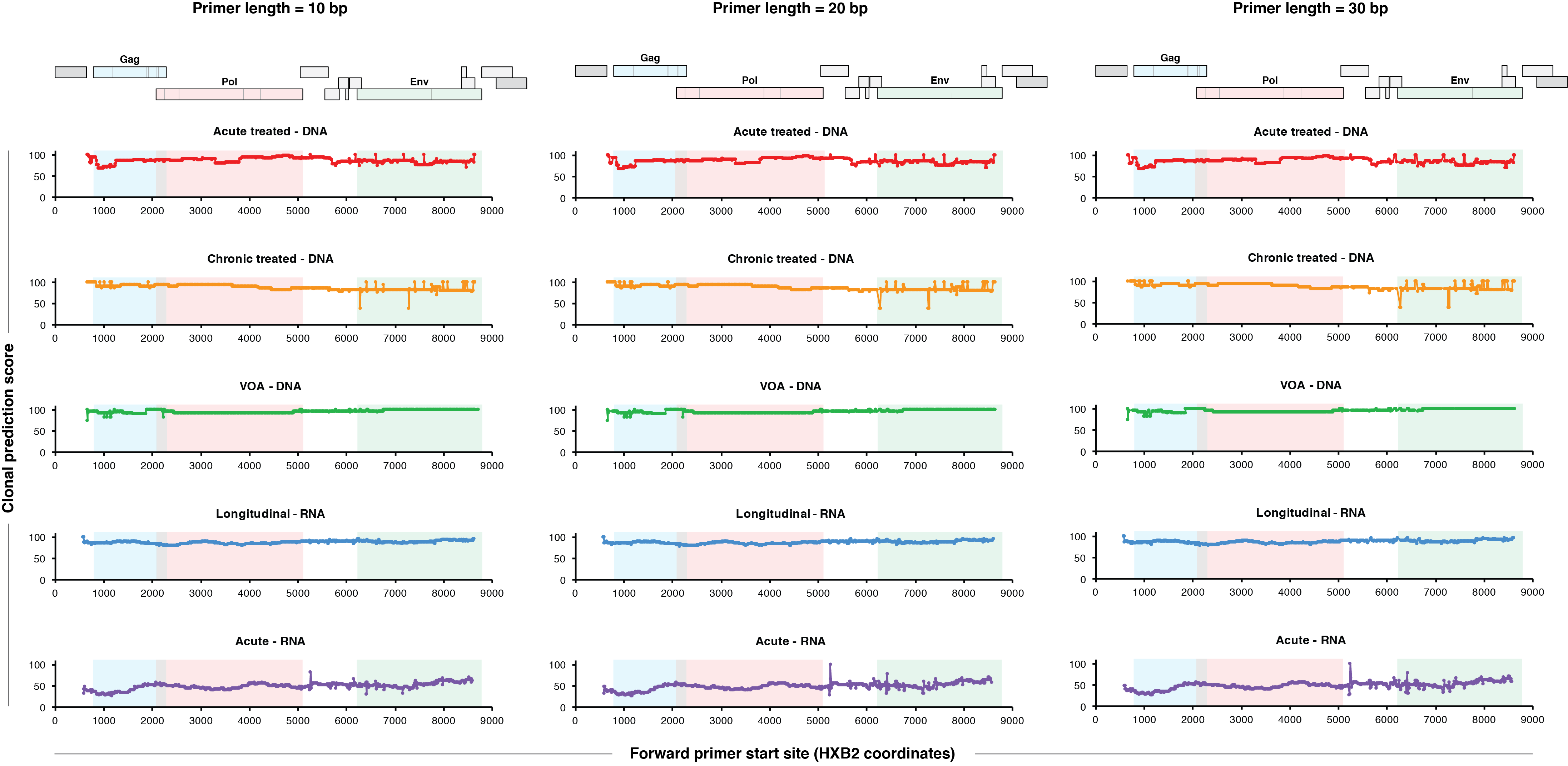

Supplement: S2 Fig — CPS values were calculated for 1 kb amplicons spanning the viral genome at 10 bp intervals. These amplicons were defined by hypothetical primers based on the HXB2 reference genome. The choice of hypothetical primer length used to define the amplicons characterized in Fig 1 is arbitrary; we show here that the results in Fig 1D are insensitive to variation in hypothetical primer length. (TIF) [file ppat.1005689.s003.tif]

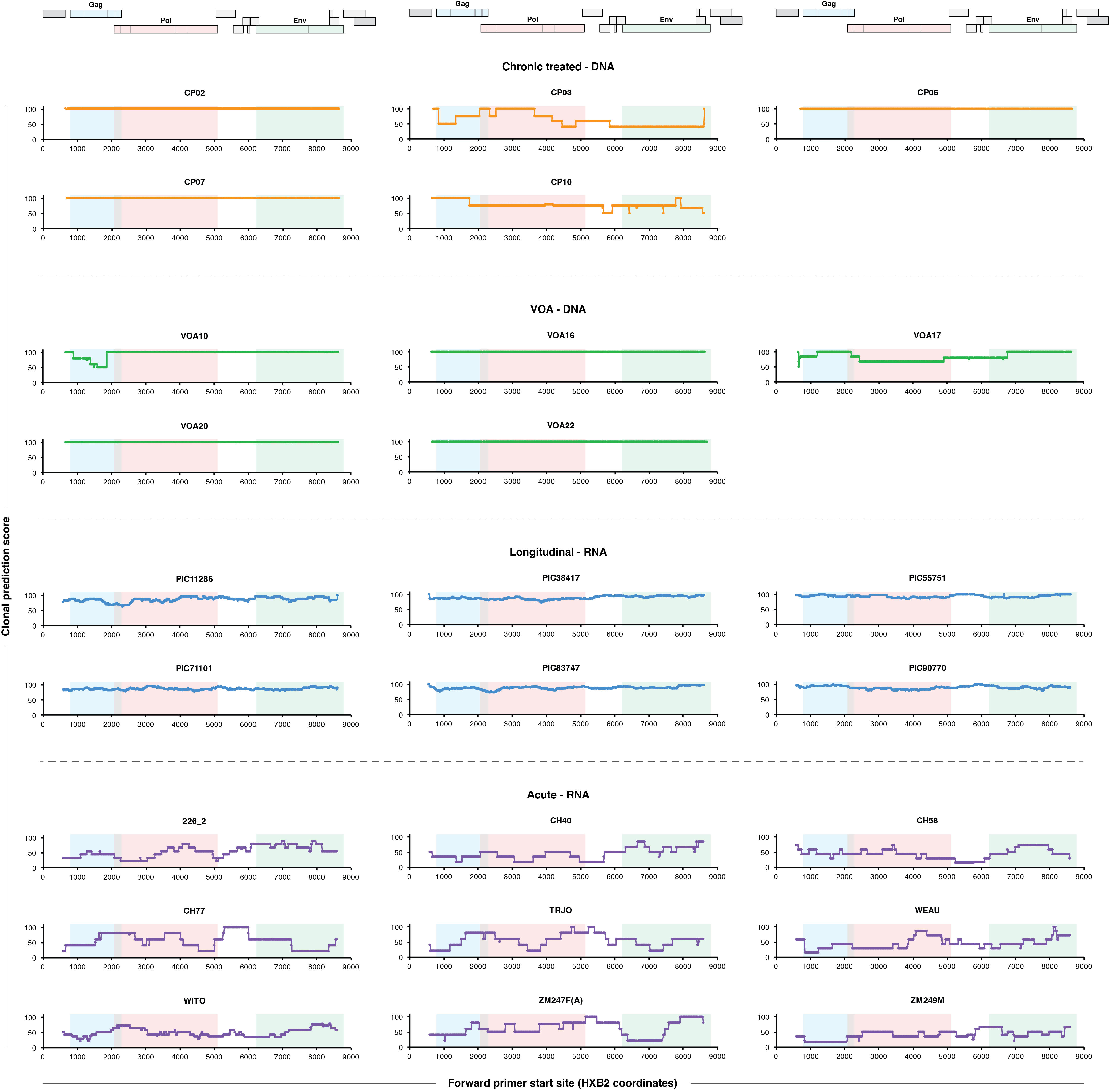

Supplement: S3 Fig — CPS of 1 kb-wide amplicons spanning the HIV-1 genome for all subjects not shown in Fig 1B. The average of these plots over all subjects within each sample type are shown in Fig 1C. (TIF) [file ppat.1005689.s004.tif]

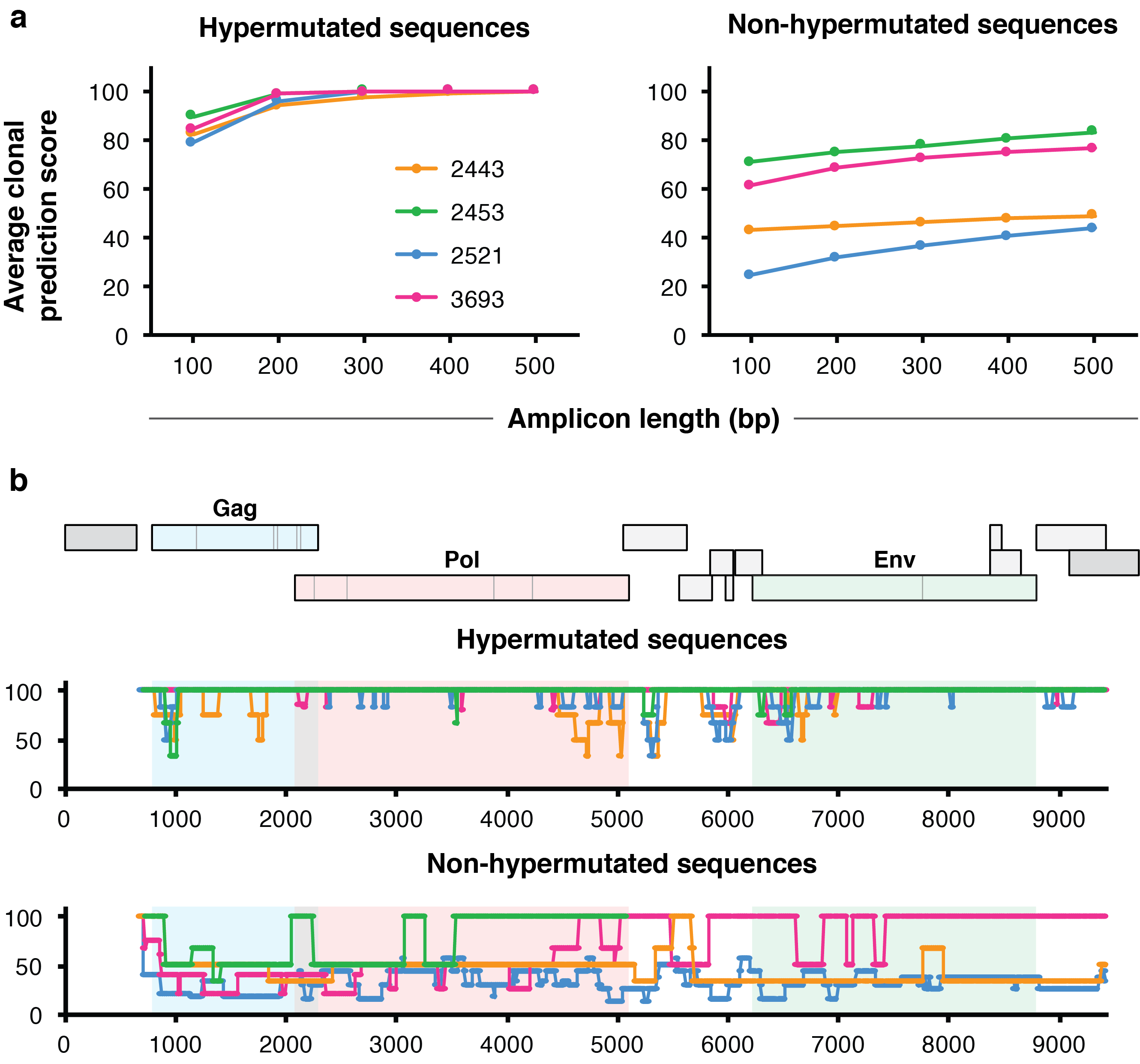

Supplement: S4 Fig — CPS values were calculated separately for the hypermutated and non-hypermutated sequences from the same subjects. Results are shown for four subjects with ≥4 unique hypermutated sequences and ≥3 unique non-hypermutated sequences. (a) Average CPS values over all hypothetical amplicons of a given length spanning the viral genome are shown for the hypermutated-only and non-hypermutated-only alignments (see Fig 2). CPS values are higher for hypermutated sequences, indicating that hypermutated sequences are much easier to distinguish than non-hypermutated sequences using amplicons as small as 100 bp. (b) CPS of 200 bp-wide amplicons spanning the HIV-1 genome (see Fig 1) with hypermutated and non-hypermutated sequences evaluated separately. The top plot emphasizes locations in the genome where even hypermutated sequences are not always distinguishable by a 200 bp amplicon. (TIF) [file ppat.1005689.s005.tif]
